# Combined analysis of the endophytic fungi and volatile oil content of different *Aquilaria sinensis* germplasms revealed the correlations between endophytic fungal abundances and agarwood production

**DOI:** 10.3389/fpls.2025.1546050

**Published:** 2025-05-13

**Authors:** Yuyin Zhang, Zixiao Jiang, Hua Dou, Chenlu Fan, Jianhe Wei, Xuyu Chen

**Affiliations:** ^1^ Hainan Provincial Key Laboratory of Resources Conservation and Development of Southern Medicine, Hainan Branch of Institute of Medicinal Plant Development, Chinese Academy of Medicinal Sciences & Peking Union Medical College, Haikou, Hainan, China; ^2^ Key Laboratory of Bioactive Substances and Resources Utilization of Chinese Herbal Medicine, Institute of Medicinal Plant Development, Chinese Academy of Medical Sciences & Peking Union Medical College, Beijing, China; ^3^ Ministry of Education & National Engineering Laboratory for Breeding of Endangered Medicinal Materials, Institute of Medicinal Plant Development, Chinese Academy of Medical Sciences & Peking Union Medical College, Beijing, China

**Keywords:** *Aquilaria sinensis*, agarwood, Qinan-type agarwood, endophytic fungi, volatile oil

## Abstract

**Introduction:**

Agarwood, a prized aromatic resin from *Aquilaria sinensis*, is formed as a defensive response to injury or fungal infection. However, the factors influencing its chemical composition remain poorly understood. This study aimed to explore the relationship between endophytic fungal communities and volatile oil content in ordinary-type and Qinan-type *A. sinensis*.

**Methods:**

Using high-throughput sequencing, we analyzed the fungal composition in both the healthy wood and agarwood layers of different *A. sinensis* germplasms. Additionally, gas chromatography-mass spectrometry (GC-MS) was employed to quantify the volatile oil content.

**Results:**

The results revealed that the fungal community composition in the agarwood layer differed between the two types of *A. sinensis*, with Fusarium, Hermatomyces, and Rhinocladiella linked to sesquiterpene production (r>0.8, p<0.01), while Microidium, Cladosporium, and Cephalotrichum were associated with chromone levels (r>0.8, p<0.01). Furthermore, the volatile oil content in Qinan-type agarwood was significantly higher than that in ordinary-type agarwood, with distinct chemical profiles observed in each germplasm.

**Discussion:**

These findings provide critical insights into the role of endophytic fungi in shaping agarwood's chemical composition and have practical implications for enhancing agarwood production in the industry. Consequently, this research has significant implications for the agarwood industry, as it enhances our understanding of how fungi influence resin quality and paves the way for improving the efficiency of agarwood induction, ultimately leading to higher-quality and more sustainable production.

## 1 Introduction

Agarwood is a resinous heartwood derived from the *Aquilaria* and *Gyrinops* species of the Thymelaeceae family and is widely popular for its distinctive aroma. Agarwood trees are found mainly in some southern provinces of China and Southeast Asia (Wang et al., 2021). Agarwood is known as the “wood of the gods” and is widely used in the cosmetics and perfume industries, medicine, religion and other fields. In China, agarwood is not only a valuable traditional spice but also a traditional Chinese medicine that has the effect of relieving pain, warming and stopping vomiting. Moreover, modern pharmacological experiments have demonstrated the sedative-hypnotic and antidepressant properties of agarwood (Wang et al., 2017; Chen et al., 2022; Zhang et al., 2019).

Under natural conditions, the resin formation process of agarwood is slow, and the resin yield is low. Physical injuries, such as lightning strikes and insect infestations, induce a defensive reaction in the tree that leads to the production of resin. It is widely believed that fungal infestation of the tree trunk after injury is involved in the formation of agarwood resin. Fungi such as *Fusarium* sp., *Aspergillus niger*, *Penicillium polonicum*, *Cladosporium* sp., and *Mucor* sp. may be involved in the process of agarwood formation (Faizal et al., 2017; Subasinghe et al., 2019; Faizal et al., 2022). Owing to the high demand for agarwood and the low yield of natural agarwood, practitioners use artificial induction methods to induce agarwood formation in trunks, such as drilling holes, infusing special fluids into the trunk, or inoculating with fungi that have a resin formation-promoting effect in the trunk (Zhang et al., 2021; Mohamed et al., 2014; Liu et al., 2022; Van Thanh et al., 2015).

*Aquilaria sinensis* (Lour.) Gilg (abbreviated as *A. sinensis*) is the source plant for agarwood in China and is divided into ordinary-type and Qinan-type *A. sinensis.* The resin formed by ordinary-type *A. sinensis* is called ordinary agarwood, and the resin formed by the Qinan-type *A. sinensis* is called Qinan agarwood. Qinan-type *A. sinensis* is a high-quality germplasm that readily bears agarwood with a high resin content, and the aroma of this resin is different from that of ordinary agarwood (Hou et al., 2022). Previously, some scholars (Hu et al., 2023) compared the chemical compositions of Qinan agarwood and ordinary agarwood and reported that the contents of phloem and ray parenchyma cells in Qinan-type germplasms were greater than those in ordinary germplasms and that the level of resin secretion in Qinan-type *A. sinensis* was much greater than that in ordinary-type *A. sinensis*. Qinan-type *A. sinensis* has a stronger photosynthetic basis for greater carbon source supply, a more efficient xylem structure for agarwood production and a higher carbon source utilization rate, leading to a higher agarwood yield and oil content (Li et al., 2022). They also confirmed that the bacterial inhibition rate of smoke from burning Qinan-type resin was slightly greater than that of burning ordinary agarwood. In long-term production, the relevant practitioners categorize different Qinan-type *A. sinensis* varieties according to parameters such as plant appearance, leaf shape, and scent properties (Yang et al., 2023). Owing to the large number of Qinan-type *A. sinensis* germplasm types and the significant differences in incense production performance, we selected three Qinan-type *A. sinensis* germplasms with superior incense production performance, which were named the Tangjie germplasm, Ruhu germplasm, and Aoshen germplasm. Ordinary-type *A. sinensis* was used as a control.

A previous study revealed differences in the abundance of endophytic fungi in different parts of agarwood (Liu et al., 2019). In 1935, Burkill (1935) reported that the formation of agarwood is the result of fungal attack and infection. Tamuli et al. (2000) isolated two strains of fungi from *A. sinensis*, inoculated the strains onto *A. sinensis* trees, and successfully induced agarwood production. Tabata et al. (2003) used five species of Fusarium to induce agarwood formation. Three fungal strains isolated by Chen’s research group, namely, *Pestalotiopsis virgatula*, *Lasiodiplodia theobromae*, and *Fomitopsis* sp., could promote the formation of agarwood (Chen et al., 2017), but there was no direct evidence of the relationship between the formation of agarwood and the fungi. In production, the formation of agarwood and the easy induction of agarwood formation in Qinan-type *A. sinensis* are considered to be related to fungi (Chen et al., 2018), but there is no evidence for this relationship. In this study, the fungi in the agarwood layer and the healthy wood layer of Qinan-type and ordinary-type *A. sinensis* trees were analyzed via high-throughput sequencing. The formation of diterpenes, chromones, fatty acid analogues and other aromatic compounds in agarwood were found to be related to fungi.

This study hypothesized that the composition of the endophytic fungal communities in the agarwood and healthy wood layers of *A. sinensis* played a significant role in determining the chemical composition of agarwood, particularly in relation to volatile oil content and specific secondary metabolites. Specifically, the study aimed to explore the correlation between the abundance and diversity of endophytic fungi and the production of key compounds such as diterpenes, chromones, and fatty acid analogues in both ordinary-type and Qinan-type *A. sinensis*. High-throughput sequencing was employed to analyze the fungal communities present in the different wood layers, and their potential influence on the formation of distinct chemical profiles of agarwood was investigated. Additionally, the research sought to compare fungal compositions and volatile oil content between different *A. sinensis* germplasms, with particular emphasis on the superior incense-producing germplasms of Qinan-type *A. sinensis*. By identifying fungal genera associated with specific compounds, this study aimed to contribute to the development of biological induction methods for high-quality agarwood production. These findings provided valuable insights into optimizing agarwood cultivation and resin induction, with potential implications for enhancing both the quality and sustainability of agarwood production in the industry.

## 2 Materials and methods

### 2.1 Study location and sampling methods

At the agarwood cultivation base of Dianbai District, Maoming city, Guangdong Province, China (110°54′E, 21°22′N), three Qinan-type *A. sinensis* germplasms, namely, the Aoshen, Tangjie and Ruhu germplasms, were selected, and the trunks of the trees were wounded via perforation to induce agarwood production. The ordinary-type *A. sinensis* germplasms in which agarwood production was induced by trunk perforation were selected as the control group. Selected trees used as experimental materials were 48 months old with an induction period of 12 months. Agarwood-bearing trees of comparable thickness and uniform growth were selected, the branches and trunks of the agarwood-bearing parts were cut, and the healthy wood and agarwood layers of the samples were scraped into small pieces using a knife for the next step of the experiment (**Supplementary Figure S1**). The different germplasm treatment groups were designated BMXH, BMXA, ASH, ASA, TJH, TJA, RHH, and RHA. BMXH and BMXA were the healthy wood layer and agarwood layer of ordinary-type *A. sinensis*, respectively; ASH and ASA were the healthy wood layer and agarwood layer of the Aoshen germplasm, respectively; TJH and TJA were the healthy wood layer and agarwood layer of the Tangjie germplasm, respectively; and RHH and RHA were the healthy wood layer and agarwood layer of the Ruhu germplasm, respectively. Aoshen, Ruhu and Tangjie are germplasms of Qinan-type *A. sinensis.*

### 2.2 DNA extraction, PCR amplification and DNA sequencing

All samples were collected from the main trunks of trees at a height of 2.0–2.5 meters above the ground. Agarwood samples were obtained by cutting approximately 5 cm below the wound area and were designated as Group A. Healthy wood samples were collected from the surrounding area of the agarwood and were labeled as Group H. After liquid nitrogen treatment, the samples were stored in a -80°C freezer for future use. Total microbial DNA from different parts of *Aquilaria sinensis* and three types of Qinan-type *A. sinensis* were extracted separately, following the method described by Ambardar et al. (2016).

The genomic DNA of each sample was extracted via the CTAB method, and then the purity and concentration of the DNA were determined via agarose gel electrophoresis. An appropriate amount of sample DNA was transferred to a centrifuge tube, and the sample was diluted with sterile water to a concentration of 1 ng/μl. The diluted genomic DNA was used as a template for PCR, and according to the selected sequencing region, specific primers with barcodes, Phusion^®^ High-Fidelity PCR Master Mix with GC Buffer from New England Biolabs, and high-fidelity enzymes were used for DNA amplification with high efficiency and accuracy. The forward primer for amplifying bacterial 16S rDNA was 799F (5′-AAC-MGGATTAGATACCCKG-3′), and the reverse primer was 1193R (5′-ACGTCATCCCCACCTTCC-3′). The forward primer for amplifying the fungal ITS1-ITS2 region was ITS1F (5′-CTTGGTCAT-TTAGAGGAAGTAA-3′), and the reverse primer was ITS2R (5′-GCTGCGTTCTTCATCGATGC-3′).

### 2.3 Sequencing data processing

The barcode and PCR amplification primer sequences from the downstream data were used to separate the data of each sample, the barcode and primer sequences were truncated via FLASH (Nguyen et al., 2016) to splice the reads of each sample, the spliced sequences were obtained as the raw reads, and fastp software was used to splice the raw reads obtained via strict filtering (Magoč and Salzberg, 2011) to obtain clean reads. The raw reads obtained from splicing were then strictly filtered via fastp software (Bokulich et al., 2013) to obtain high-quality reads (clean reads). The reads obtained after the above processing were further processed to remove chimeric sequences, and the read sequences were compared with the germplasm annotation database (Rognes et al., 2016) to detect chimeric sequences. The chimeric sequences were ultimately removed (JF et al., 2011) to obtain the final data set.

### 2.4 OTU clustering and germplasm annotation

The Uparse algorithm (Edgar, 2013) was used to cluster all effective tags of all samples, and by default, the sequences were clustered into operational taxonomic units (OTUs) with 97% similarity. At the same time, representative sequences of the OTUs were selected, and on the basis of the algorithm, the sequence with the highest frequency of occurrence among the OTUs was selected as the representative OTU sequence. Germplasm annotation of the OTU sequences was performed using the blast method (Altschul et al., 1990) in QIIME software (version 1.9.1) and the Unite (v8.2) database (Kõljalg et al., 2013), and the sequences were analyzed at each taxonomic level: kingdom, phylum, class, order, family, and germplasm to determine the community composition of each sample. A rapid multiple-sequence comparison was performed via MUSCLE software to obtain the phylogenetic relationships of all the OTUs that represented sequences. Finally, the data of each sample were homogenized by taking the least amount of data in the sample as the criterion for homogenization, and the subsequent alpha diversity analysis and beta diversity analysis were based on the homogenized data (Edgar, 2004).

### 2.5 Detection of volatile components

The original-type material used in the experiment was separated from the agarwood layer and healthy wood layer with a knife, ground to powder, and passed through a 50-mesh sieve. Then, 0.5 g of sample powder was weighed out, placed in the sample bottle, and moistened with 3 drops of pure water with a dropper. The sample was placed in a water bath at 85°C and heated for 30 min, after which the solid-phase microextraction apparatus was activated for 30 min at 240°C at the inlet port. After the activation was completed, the sample bottle was inserted, and finally, the water bath was held at 85°C to keep the quartz fibers in the steam of the aroma components. The solid-phase microextraction apparatus was removed and connected to the gas-phase mass spectrometer for analysis of the sample after 30 min of adsorption.

An HP-5MS flexible quartz capillary column (30 mm*0.25 mm, 0.25 μm) was used, the carrier gas was high-purity helium, the volumetric flow rate was 1 ml/min, the inlet temperature was 250°C, and the detector temperature was 300°C. After the column temperature was maintained at 50°C for 1 min, the temperature was increased to 143°C at a rate of 15°C/min, maintained for 10 min, increased to 155°C at a rate of 1°C/min, increased to 225°C at a rate of 25°C/min, maintained for 7 min, increased to 250°C at a rate of 25°C/min, and finally maintained for 10 min.

The electron bombardment (EI) energy was 70 eV, the ion source temperature was 250°C, the solvent delay was 5 min, and the scanning range was 50–500 amu.

### 2.6 Data analysis

The means and standard errors of all the data were calculated, and all the values are reported as the means of three replicates. Venn diagrams were drawn to analyze the shared and unique OTUs between sample groups on the basis of the OTUs obtained from clustering and the research needs. The fungal community richness and diversity indices of individual germplasm samples were analyzed via differences in the alpha diversity index. Fungal distribution at the genus level was analyzed via heatmap analysis. Differences in the volatile contents of different agarwood samples were analyzed via principal component analysis (PCA). In addition, the relationships between fungal communities and volatile components were analyzed via Pearson correlation analysis. All the statistical analyses were performed via SPSS v. 16.0 (Kõljalg et al., 2013).

## 3 Results

### 3.1 High-throughput sequencing of endophytic fungi

**Figure 1:**
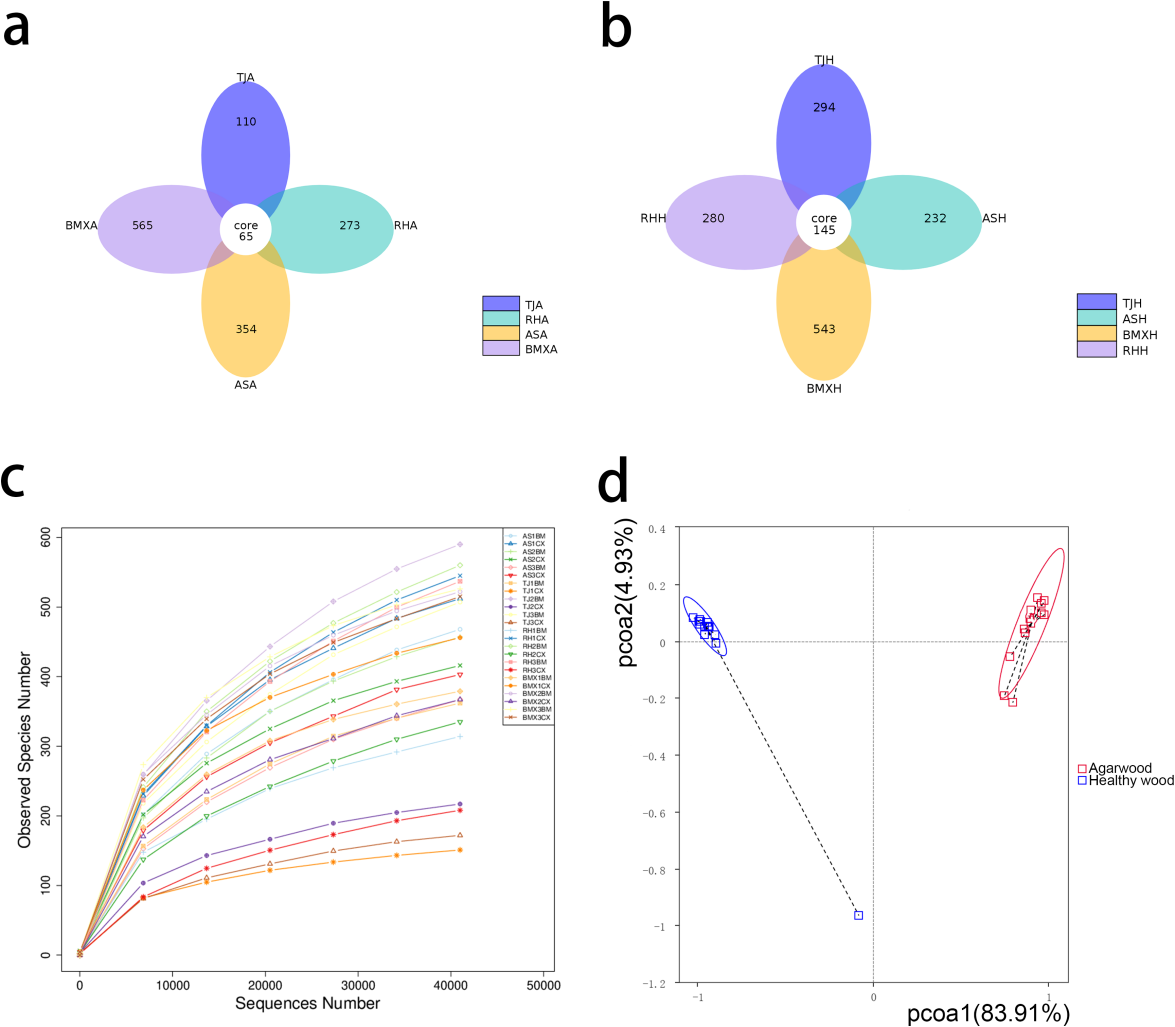
Analysis of fungal diversity in agarwood and healthy wood samples (n= 3). **(a)** Venn diagram of OTUs in the agarwood groups; **(b)** Venn diagram of OTUs in the healthy wood groups; **(c)** rarefaction curves for observed species in all samples; **(d)** PCoA diagram of fungal compositions in all samples.

A total of 44,194~88,107 qualified reads were obtained from the agarwood layer and healthy wood layer of *A. sinensis* (ordinary-type and Qinan-type) trees, belonging to 15 phyla, 56 classes, 162 orders, 375 families and 766 genera, and 178~645 OTUs were identified (**Supplementary Table S1**), with a similarity of 97%. Venn diagram analysis revealed that there were only 65 shared OTUs in the agarwood layer of each *A. sinensis* germplasm, which was much fewer than the number of unique OTUs in each group, and there were 146 shared OTUs in the healthy wood layer. The number of specific OTUs in the three kinds of Qinan agarwood were 110, 273, and 354. The number of specific OTUs in the agarwood layer and healthy wood layer of ordinary-type *A. sinensis* were 565 and 543, respectively, which were greater than those in Qinan-type *A. sinensis* (**Figures 1a, b**). As the number of sampled sequences increased, the rarefaction curves for all samples were approximately parallel to the x-axis, indicating that the sequencing depth used in this experiment was reliable (**Figure 1c**). PCoA revealed that the fungal compositions of the agarwood layer and the healthy wood layer were distinguishable between Qinan-type and ordinary-type *A. sinensis* (**Figure 1d**).

Five alpha diversity indices were used to analyze the richness, diversity, and coverage of fungal microbiomes in the samples. The Good’s coverage was greater than 99.6%, indicating that the sampling depth met the requirements for analysis. The ACE and Chao1 indices of each agarwood group were greater than those of the healthy wood group of the same germplasm, and the fungal abundance was greater. Except for those of the Ruhu germplasm, the Shannon and Simpson indices of the agarwood layers of the germplasms were greater than those of the healthy wood layers, and the fungal diversity was greater (**Table 1**). These findings suggest that fungal communities in the agarwood layers exhibit greater richness and diversity than in the healthy wood layers, likely due to the distinct environmental and biochemical conditions associated with agarwood formation. The higher fungal diversity, particularly in *A. sinensis* germplasms such as Aoshen, Tangjie, and ordinary-type, supports the idea that the microbial environment significantly influences the chemical composition and quality of agarwood.

**Table 1:** Alpha diversity indices of the samples.

Samples	Chao1	Ace	Simpson	Shannon	Goods coverage
BMXA	560.231	580.76	0.616	2.646	0.997
ASA	548.406	580.694	0.684	2.946	0.997
RHA	541.695	566.812	0.311	1.428	0.996
TJA	230.756	234.087	0.367	1.435	0.999
BMXH	578.322	593.492	0.293	1.697	0.997
ASH	598.709	620.176	0.226	1.283	0.996
RHH	629.046	658.658	0.403	1.912	0.996
TJH	661.604	699.929	0.22	1.306	0.996

### 3.2 Composition of fungi in agarwood layers of ordinary-type and Qinan-type *Aquilaria sinensis*

**Figure 2:**
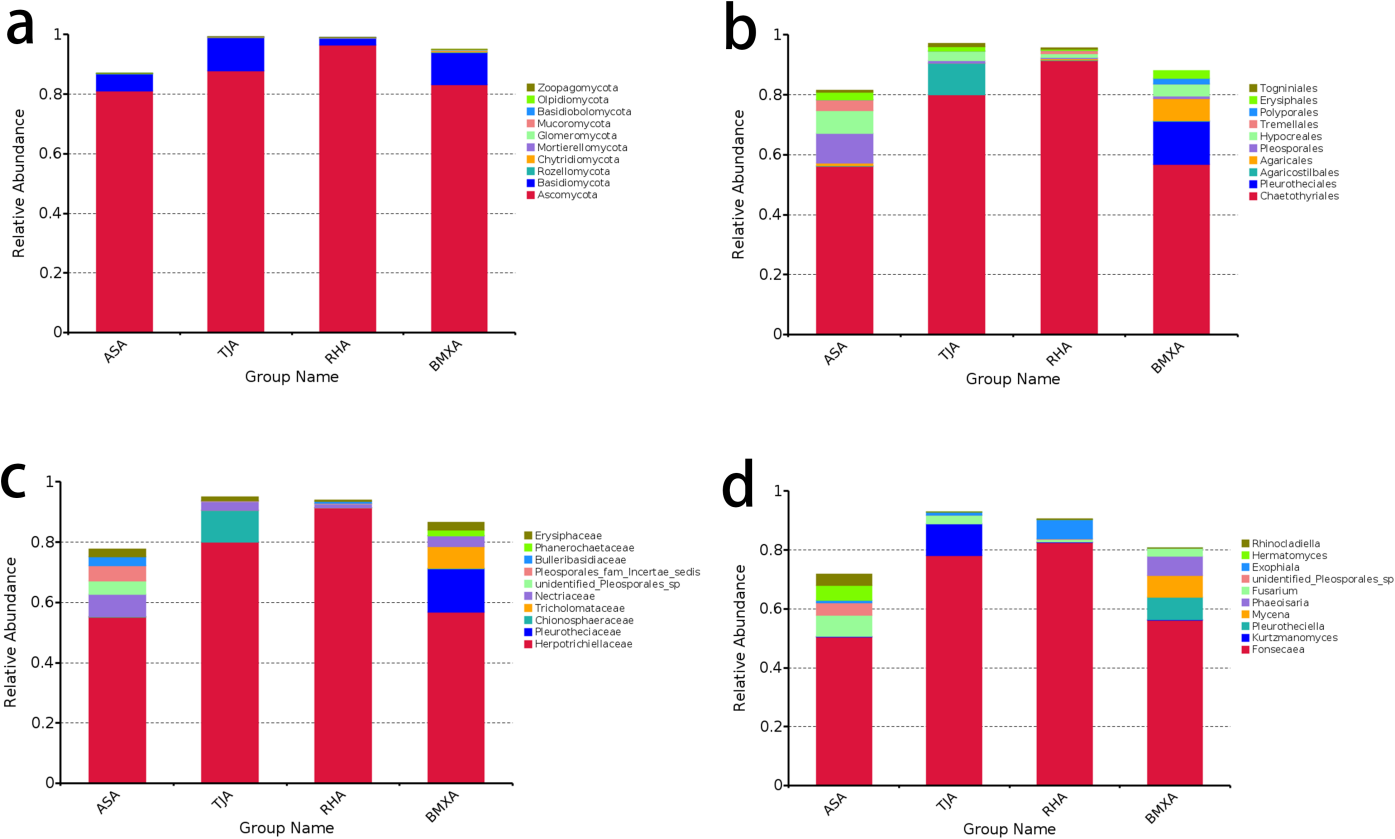
Fungal composition of the agarwood samples of ordinary-type and Qinan-type *A. sinensis* at the phylum **(a)**, order **(b)**, family **(c)**, and genus **(d)** levels (n= 3).

The dominant phylum in the agarwood layers of Qinan-type and ordinary-type *A. sinensis* was Ascomycota, which accounted for more than 80% of the taxa in all the groups, followed by Basidiomycota, which accounted for approximately 5%-18%, with other phyla exhibiting lower abundances (**Figure 2a**). At the order classification level, the dominant order in Qinan-type and ordinary-type *A. sinensis* was Chaetothyriales, with Pleosporales and Hypocreales showing proportions of 10% in the Aoshen germplasm and the remaining orders showing lower proportions. of the proportion of Agaricostilbales in the Tangjie germplasm was approximately 12%, and that of Pleurotheciales was approximately 14% in ordinary-type *A. sinensis* (**Figure 2b**).

At the family classification level, *Herpotrichiellaceae* was the dominant family in Qinan-type and ordinary-type *A. sinensis*, with *Herpotrichiellaceae* accounting for approximately 52% in the Aoshen germplasm, followed by Nectriaceae at approximately 9%; *Herpotrichiellaceae* accounted for 80% in the Tangjie germplasm, followed by *Chionosphaeraceae* with 10%; *Herpotrichiellaceae* accounted for 92% in the Ruhu germplasm, and the remaining families accounted for a lower percentage; *Herpotrichiellaceae* accounted for 80% in the Tangjie germplasm, followed by *Chionosphaeraceae* with 10%; *Herpotrichiellaceae* accounted for 92% in the Ruhu germplasm, and the remaining families accounted for a lower percentage; *Herpotrichiellaceae* accounted for 58% in ordinary-type *A. sinensis*; and *Pleurotheciaceae* accounted for 19% in the germplasm (**Figure 2c**). Further categorization revealed that the dominant genus in each species was *Fonsecaea*, which accounted for 49% in the Aoshen germplasm, with the remaining genera, *Fusarium* and *Heimatomyces*, accounting for 9% and 7%, respectively; *Fonsecaea* accounted for 78% of the species in the Tangjie germplasm; *Kurtzmanomyces* accounted for 10%; and the remaining genera accounted for a lower percentage; *Fonsecaea* accounted for 81% of the species in the Ruhu germplasm, and *Exophiala* accounted for 8%; *Fonsecaea* accounted for 56% of the species in ordinary-type *A. sinensis*, while *Pleurotheciella*, *Mycena* and *Phaeoisaria* each accounted for approximately 8%(**Figure 2d**).

### 3.3 Composition of fungal microbiomes in healthy wood layers of ordinary-type and Qinan-type *Aquilaria sinensis*

**Figure 3:**
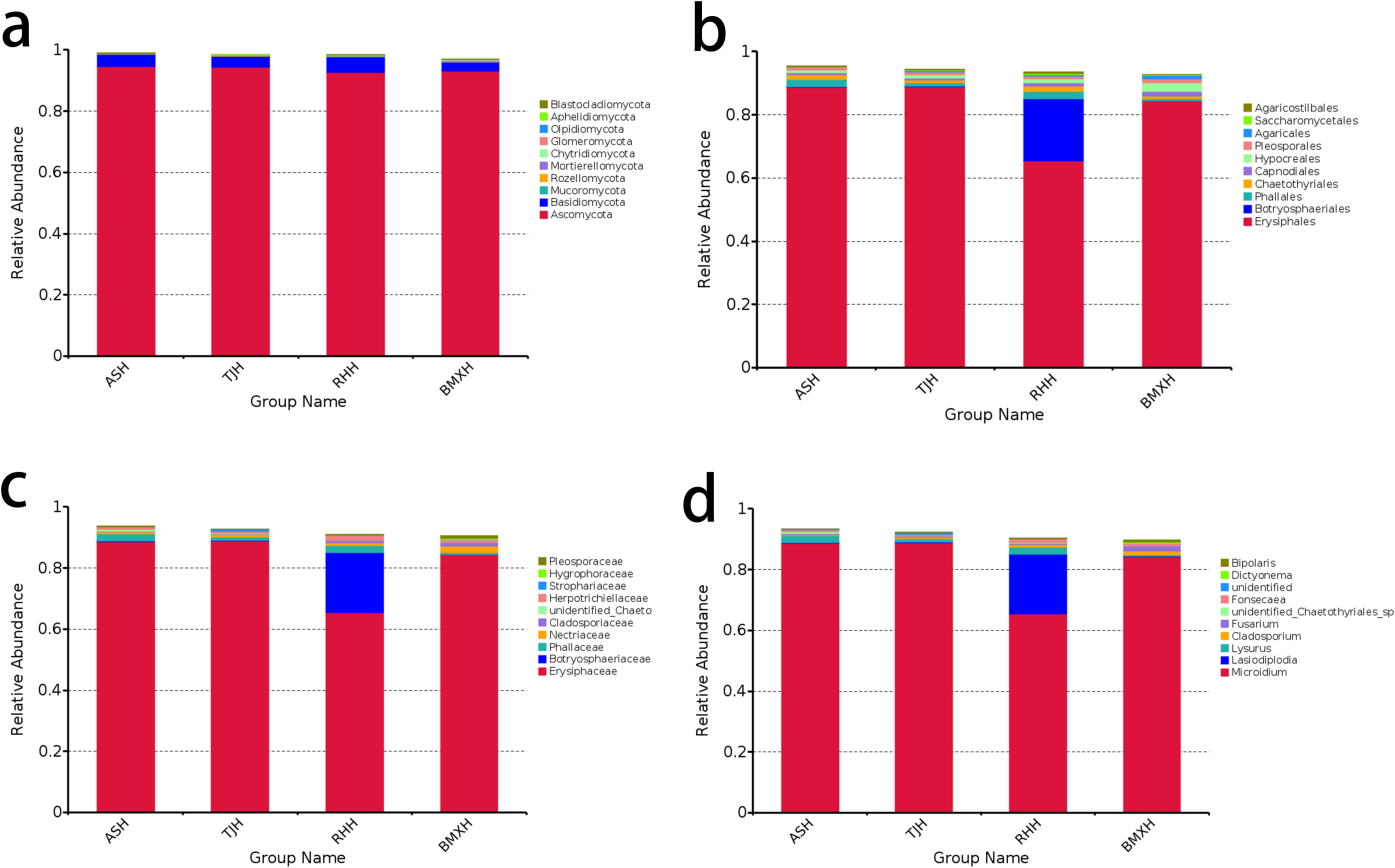
Fungal composition of the healthy wood samples of ordinary-type and Qinan-type *A. sinensis* at the phylum **(a)**, order **(b)**, family **(c)**, and genus **(d)** levels (n= 3).

In the healthy wood groups of the four germplasms, the dominant phylum was Ascomycota, accounting for more than 90%, followed by Basidiomycota, accounting for approximately 3%, while the remaining phyla accounted for a relatively small proportion (**Figure 3a**). At the order classification level, *Erysiphales* was the dominant order, accounting for approximately 84% in the Aoshen, Tangjie and ordinary-type *A. sinensis* germplasms, whereas the remaining orders accounted for lower proportions. *Leotiomycetes* accounted for 64% in the Ruhu germplasm, and *Botryosphaeriales* accounted for approximately 20% (**Figure 3b**). At the family classification level, *Erysiphaceae* was the dominant family, accounting for approximately 84% in the Aoshen, Tangjie and ordinary-type *A. sinensis* germplasms. *Erysiphaceae* accounted for 64% and *Botryosphaeriaceae* accounted for 20% in the Ruhu germplasm (**Figure 3c**). At the genus classification level, *Microidium* was the dominant genus, accounting for approximately 84% in the Aoshen, Tangjie and ordinary-type *A. sinensis* germplasms. *Erysiphaceae* accounted for 64% and *Botryosphaeriaceae* accounted for 20% in the Ruhu germplasm (**Figure 3d**).

The histogram of fungal classification at the genus level (**Figure 2d**, **Figure 3d**) revealed that the dominant genus in the healthy wood layer of the four types *A. sinensis* was *Microidium*, and the dominant genus in the agarwood layer was *Fonsecaea*. According to the analysis of the correlations between each fungal genus and the volatile components, the abundance of *Fonsecaea* was correlated with the levels of a variety of volatile components (**Table 2**). The differences in the distribution of the fungal genera between the healthy wood layers of each germplasm were not apparent, and the differences in the distribution of the fungal genera among the agarwood layers of the four germplasms were more notable. Although the dominant genus in each germplasm was *Fonsecaea*, there were differences in the distribution in each germplasm in terms of the abundances of the top genera. Several of the higher-ranking fungi in the three Qinan agarwood layers, such as *Fusarium*, *Vanakripa*, *Hermatomyces* and *Aspergillus*, were found at extremely low levels in ordinary agarwood, whereas the correlation analysis revealed that the top-ranked genera were significantly related to the formation of chromones. The analysis of the volatile component content also revealed that the chromone content in the three Qinan agarwood layers was much greater than that in the ordinary agarwood.

**Table 2:** Correlations between volatile compounds and fungal genera (n= 3).

Chemical class	Compounds	Molecular formula	Relative molecular mass	Fungal genus	r-value
Diterpenes	Ylangene	C_15_H_24_	204.1878	*Myrothecium*	0.898**
				*Fonsecaea*	0.935**
				*Exophiala*	0.874**
				*Veronaea*	0.898**
				*Fonsecaea*	0.974**
				*Trichosporon*	0.972**
				*Conioscypha*	0.858**
	β-Elemene	C_15_H_24_	204.35	*Kurtzmanomyces*	0.960**
				*Arthopyrenia*	0.839**
				*Trichosporon*	0.945**
	α-Cyperene	C_15_H_24_	204.1878	*Conioscypha*	0.966**
	α-Bergamotene	C_15_H_24_	204.35	*Kurtzmanomyces*	0.999**
				*Arthopyrenia*	0.909**
	α-Guaiene	C_15_H_24_	204.35	*Trichosporon*	0.935**
				*Conioscypha*	0.972**
				*Kurtzmanomyces*	0.999**
				*Arthopyrenia*	0.909**
	Sativene	C_15_H_24_	204.1878	*Trichosporon*	0.935**
				*Conioscypha*	0.972**
				*Kurtzmanomyces*	0.999**
				*Arthopyrenia*	0.909**
	β-Patchoulene	C_15_H_24_	204.1878	*Trichosporon*	0.935**
				*Conioscypha*	0.972**
				*Veronaea*	0.877**
				*Fonsecaea*	0.904**
	Humulene	C_15_H_24_	204.35	*Fonsecaea*	0.835**
				*Exophiala*	0.843**
				*Veronaea*	0.859**
	Cypera-2,4-diene	C_15_H_22_	202.33	*Fonsecaea*	0.944**
	α-Curcumene	C_15_H_22_	202.33	*Kurtzmanomyces*	0.944**
	β-Selinene	C_15_H_24_	204.35	*Trichosporon*	0.950**
				*Conioscypha*	0.942**
				*Fonsecaea*	0.896**
	Valencene	C_15_H_24_	204.35	*Fusarium*	0.917**
	α-Selinene	C_15_H_24_	204.35	*Hermatomyces*	0.868**
	α-Bulnesene	C_15_H_24_	204.35	*Rhinocladiella*	0.860**
				*Vanakripa*	0.859**
				*Pseudeurotium*	0.838**
	Cadina-1(10),4-diene	C_15_H_24_	204.35	*Stachybotrys*	0.836**
	Cadala-1(10),3,8-triene	C_15_H_22_	202.33	*Stachybotrys*	0.993**
				*Fusarium*	0.906**
				*Hermatomyces*	1.000**
				*Rhinocladiella*	0.999**
				*Derxomyces*	0.971**
				*Vanakripa*	0.999**
	Kessane	C_15_H_26_O	222.37	*Pseudeurotium*	0.919**
				*Chaetomium*	0.844**
				*Stachybotrys*	0.981**
				*Fusarium*	0.925**
				*Hermatomyces*	0.978**
				*Rhinocladiella*	0.979**
				*Derxomyces*	0.979**
				*Vanakripa*	0.977**
	α-Calacorene	C_15_H_2_0	200.32	*Pseudeurotium*	0.931**
				*Chaetomium*	0.885**
				*Exophiala*	0.983**
				*Veronaea*	0.958**
				*Fonsecaea*	0.942**
				*Hermatomyces*	0.974**
				*Rhinocladiella*	0.972**
	Sesquirosefuran	C_15_H_22_O	218.33	*Derxomyces*	0.933**
				*Vanakripa*	0.971**
	9,10-dehydro-Isolongifolene	C_15_H_2_0	200.32	*Pseudeurotium*	0.864**
	α-Santalol	C_15_H_24_O	220.35	*Chaetomium*	0.856**
	1-Methyl-4-methylene-2-(2-methyl-1-propenyl)-1-vinylcycloheptane	C_15_H_24_	204.35	*Stachybotrys*	0.983**
				*Stachybotrys*	0.994**
				*Hermatomyces*	1.000**
				*Rhinocladiella*	1.000**
				*Derxomyces*	0.972**
				*Vanakripa*	1.000**
				*Pseudeurotium*	0.917**
	α-Gurjunene	C_15_H_24_	204.35	*Chaetomium*	0.854**
				*Exophiala*	0.867**
				*Veronaea*	0.873**
				*Lasiodiplodia*	0.958**
				*Kurtzmanomyces*	0.999**
				*Arthopyrenia*	0.909**
				*Trichosporon*	0.935**
	Spathulenol	C_15_H_24_O	220.35	*Conioscypha*	0.972**
				*Fonsecaea*	0.961**
	epi-Eudesmol	C_15_H_26_O	222.37	*Fonsecaea*	0.881**
	γ-Eudesmole	C_15_H_26_O	222.37	*Fonsecaea*	0.949**
	Agarospirol	C_15_H_26_O	222.37	*Exophiala*	0.834**
	Guaia-9,11-diene	C_15_H_24_	204.35	*Veronaea*	0.843**
	Cadinene	C_15_H_26_	206.37	*Fonsecaea*	0.988**
	α-Eudesmol	C_15_H_26_O	222.37	*Fonsecaea*	0.995**
	Neointermedeol	C_15_H_26_O	222.37	*Fonsecaea*	0.857**
	3,5,11-Eudesmatriene	C_15_H_22_	202.33	*Exophiala*	0.948**
				*Veronaea*	0.985**
				*Candida*	0.884**
	γ-Elemene	C_15_H_24_	204.35	*Kernia*	0.835**
	Valerenol	C_15_H_24_O	220.35	*Puccinia*	0.873**
	trans-Farnesol	C_15_H_26_O	222.37	*Epicoccum*	0.876**
				*Fonsecaea*	0.860**
	Alloaromadendrene oxide-(2)	C_15_H_24_O	220.35	*Fonsecaea*	0.887**
				*Exophiala*	0.855**
				*Veronaea*	0.870**
	5(1H)-Azulenone,2,4,6,7,8,8a-hexahydro-3,8-dimethyl-4-(1-methylethylidene)-, (8S0-cis)-	C_15_H_22_O	218.33	*Kurtzmanomyces*	0.999**
				*Arthopyrenia*	0.909**
	Isoaromadendrene epoxide	C_15_H_24_O	220.35	*Trichosporon*	0.935**
	6-Isopropenyl-4,8a-dimethyl-3,5,6,7,8,8a-hexahydro-2(1H)-naphthalenone	C_15_H_22_O	218.33	*Conioscypha*	0.972**
				*Fusarium*	0.898**
				*Hermatomyces*	1.000**
	β-Gurjunene	C_15_H_24_	204.35	*Rhinocladiella*	1.000**
				*Derxomyces*	0.972**
				*Vanakripa*	1.000**
				*Pseudeurotium*	0.917**
	Caryophyllane	C_15_H_28_	208.32	*Chaetomium*	0.854**
				*Stachybotrys*	0.994**
				*Fonsecaea*	0.969**
				*Kurtzmanomyces*	0.999**
				*Arthopyrenia*	0.909**
				*Trichosporon*	0.935**
				*Conioscypha*	0.972**
				*Veronaea*	0.958**
	Solavetivone	C_15_H_22_O	218.33	*Fonsecaea*	0.978**
	Ledol	C_15_H_26_O	222.37	*Fonsecaea*	0.899**
				*Candida*	0.884**
				*Kernia*	0.835**
	Ledene	C_15_H_24_	204.35	*Kurtzmanomyces*	0.950**
	Aromandendrene	C_15_H_24_	204.35	*Trichosporon*	0.857**
	Valerenic acid	C_15_H_22_O_2_	234.33	*Conioscypha*	0.920**
	3,5-Di-tert0butyl-2-hydroxybenzaldehyde	C_15_H_22_O_2_	234.33	*Exophiala*	0.905**
				*Veronaea*	0.845**
				*Bipolaris*	0.867**
	Eudesma-1,4(15),11-triene	C_15_H_22_	202.33	*Alternaria*	0.855**
				*Exophiala*	0.905**
				*Veronaea*	0.845**
	Velleral	C_15_H_20_O_2_	232.32	*Bipolaris*	0.867**
				*Apiotrichum*	0.897**
	8,9-dehydro-cycloisolongifolene	C_15_H_22_	202.33	*Alternaria*	0.855**
				*Exophiala*	0.905**
	Velleral	C_15_H_20_O_2_	232.32	*Veronaea*	0.845**
				*Bipolaris*	0.867**
Other aromatics	Nonanal	C_9_H_18_O	142.14	*Cephalotrichum*	0.877**
				*Solicoccozyma*	0.852**
	Decanal	C_10_H_20_O	156.15	*Naganishia*	0.928**
				*Solicoccozyma*	0.983**
				*Stachybotrys*	0.931**
	14-Octadecenal	C_18_H_34_O	266.26	*Trichosporon*	0.837**
	10,11-Epoxycalamenene	C_15_H_20_O	216.32	*Kurtzmanomyces*	0.902**
				*Sarocladium*	0.912**
	2,4,6-trimethyl0Phenol	C_9_H_12_O	136.19	*Exophiala*	0.983**
				*Veronaea*	0.958**
				*Naganishia*	0.845**
	(cyclohexyl)(2,3-dimethylphenyl)-Methanol	C_15_H_22_O	218.33	*Kurtzmanomyces*	0.999**
				*Arthopyrenia*	0.909**
				*Sarocladium*	0.935**
	(Z)-13-Octadecenal	C_18_H_34_O	266.50	*Microidium*	0.994**
Chromones	2-Methyl-4-chromone	C_10_H_8_O_2_	160.17	*Fonsecaea*	0.845**
	2,3,3-Trimethyl-2-(3-methyl-buta-1,3-dienyl)-cyclohexanone	C_14_H_22_O	206.32	*Sarocladium*	0.859**
	Spiro[1,3,30trimethylindoline]-2,5’-pyrrolidin-2-one	C_14_H_18_N_2_O	230.31	*Microidium*	0.841**
	2-(2-Phenylethyl)Chromone	C_17_H_14_O_2_	250.29	*Fusarium*	0.860**
				*Hermatomyces*	0.864**
				*Rhinocladiella*	0.862**
				*Derxomyces*	0.909**
				*Vanakripa*	0.858**
				*Pseudeurotium*	0.956**
				*Aspergillus*	0.928**
	2-(4-Methoxyphenethyl)-4H-chromen-4-one	C_18_H_16_O_3_	280.30	*Fusarium*	0.871**
				*Hermatomyces*	0.924**
				*Rhinocladiella*	0.925**
				*Derxomyces*	0.973**
				*Vanakripa*	0.922**
				*Pseudeurotium*	0.983**
				*Aspergillus*	0.980**
Fatty acid analogs	Pentadecanoic acid	C_15_H_30_O_2_	242.40	*Microidium*	0.918**
				*Gibberella*	0.838**
				*Kernia*	0.864**
	Hexadecanoic acid, methyl ester	C_17_H_34_O_2_	270.50	*Microidium*	0.960**
	n-Hexadecanoic acid	C_16_H_32_O_2_	256.42	*Cladosporium*	0.877**
				*Bipolaris*	0.886**
	Hexadecanoic acid, ethyl ester	C_18_H_36_O_2_	284.50	*Cephalotrichum*	0.967**
	Heptadecanoic acid	C_17_H_34_O_2_	270.50	*Bipolaris*	0.835**
	(Z)-6-Octadecenoic acid	C_18_H_34_O_2_	282.50	*Cladosporium*	0.899**
				*Bipolaris*	0.997**
				*Naganishia*	0.935**
				*Solicoccozyma*	0.874**
				*Stachybotrys*	0.938**
	Oleic Acid	C_18_H_34_O_2_	282.50	*Lysurus*	0.989**
				*Lamelloclavaria*	0.928**
	Octadecanoic acid	C_18_H_36_O_2_	284.50	*Lysurus*	0.936**
				*Lamelloclavaria(*	0.978**
	(E)-9-Octadecenoic acid ethyl ester	C_20_H_38_O_2_	310.50	*Cephalotrichum*	0.893**

**Correlation is significant at the 0.01 level (2-tailed).

These findings highlight the crucial role of specific fungal genera, particularly *Fonsecaea* and *Fusarium*, in shaping the chemical profile of agarwood. The results suggest that the presence of certain fungi in the agarwood layers may be instrumental in the production of key secondary metabolites, such as chromones. These insights enhance our understanding of microbial influences on agarwood quality and may contribute to the development of more effective biological induction methods for producing high-quality agarwood.

### 3.4 Comparison of the chemical compositions of different *Aquilaria sinensis* germplasms

**Figure 4:**
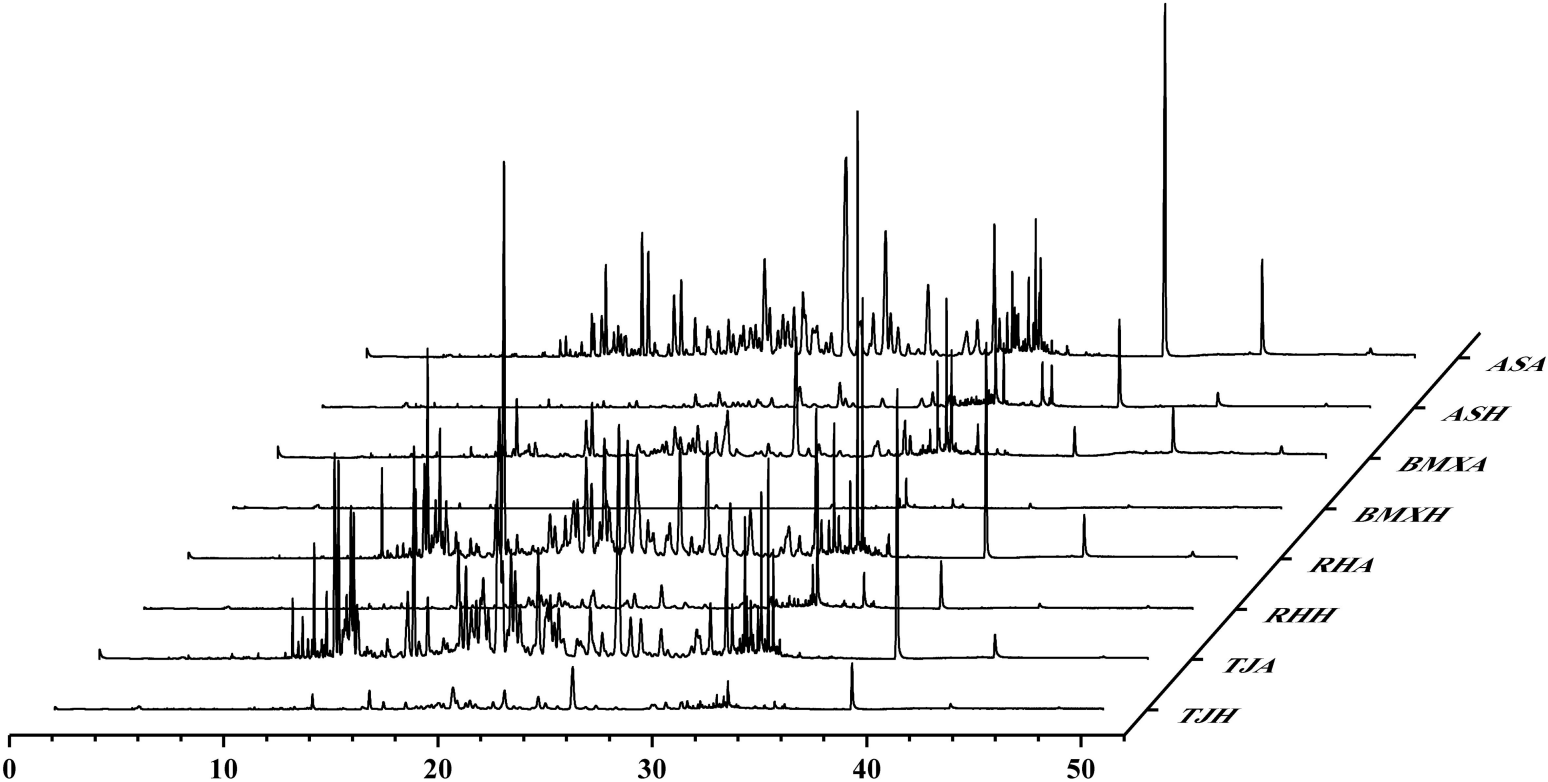
GC–MS total ion flow diagrams of 8 types of raw material components (n= 3).

NIST SEARCHER 2.4 combined with the NIST17 database was used to search for the composition of each raw agarwood material determined via GC–MS, and only compounds with a match of 50 or more were retained, resulting in a total of 126 volatile constituents: 15 other aromatic compounds, 67 sesquiterpenes (SESs), 12 fatty acid compounds (ADSs), and 13 chromone constituents. The highest number of compounds detected in TJA contained a total of 86 different chemical constituents, followed by 75 in BMXA and 71 in RHA, in contrast to ASA, which had the lowest number of constituents detected in the agarwood layer group, with only 68 in the eight raw material fractions examined (**Figure 4**). The four healthy wood layer groups were very similar in composition, whereas the agarwood layer groups all differed in terms of overall peak shape. The differences in the number, types and relative contents of chemical components between the agarwood and healthy wood layers of different germplasms were also very large.

**Figure 5:**
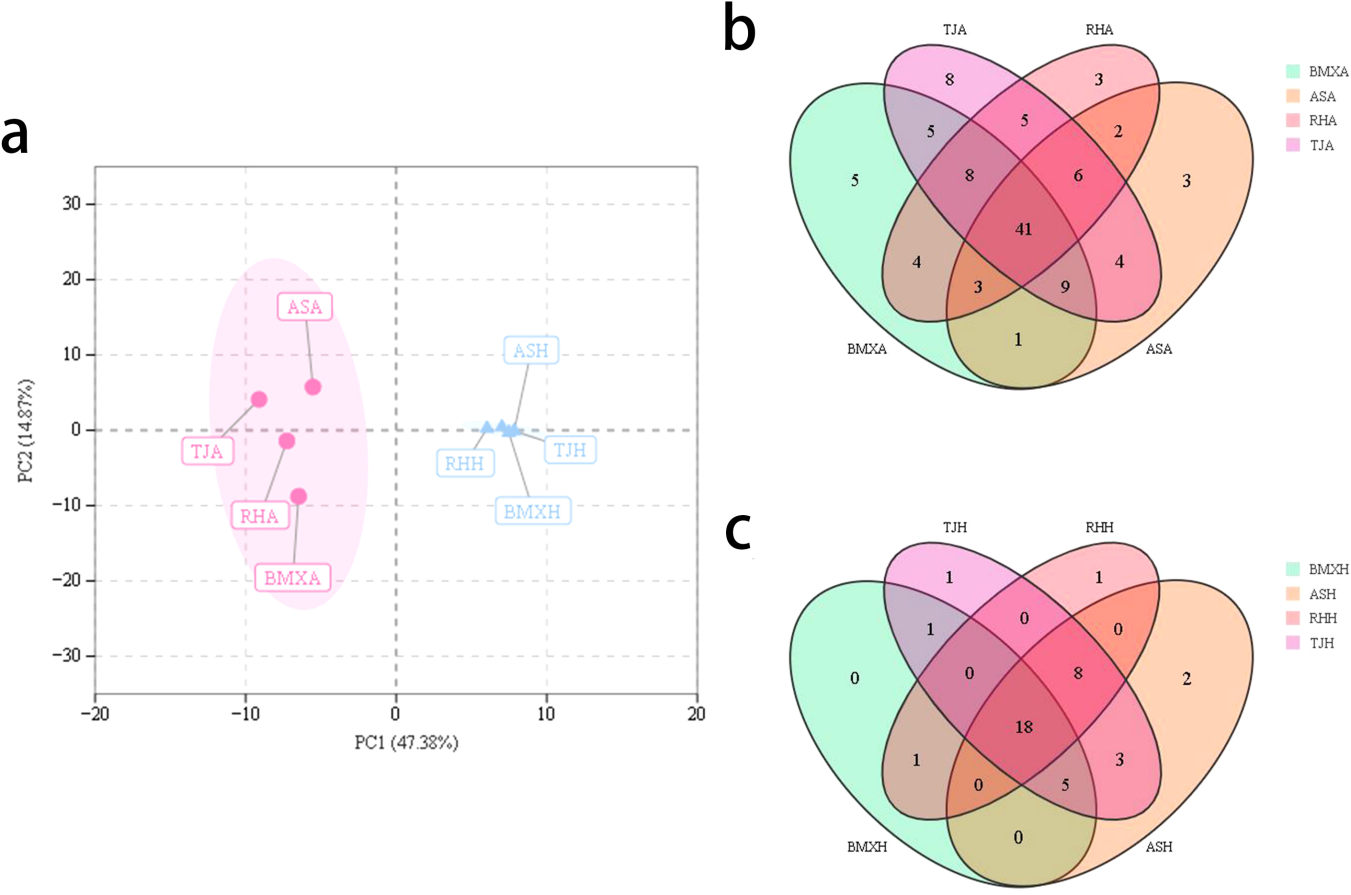
Comparison of chemical components among different groups. **(a)** Principal component analysis; **(b)** Venn diagram of the composition of the agarwood layer; **(c)** Venn diagram of healthy wood layer composition (n= 3).

To explore the relationships between intergroup and intragroup differences and the differences between the agarwood and healthy wood layers of different *A. sinensis* germplasms, we used principal component analysis (PCA) plots. The first principal component t[1] accounted for 47.38% of the sample variance, and the second principal component t[2] accounted for 14.87% of the sample variance. The results revealed that the differences between the two groups of agarwood and the healthy wood layer were very large, whereas the differences within the groups were relatively small. The PCA plots clearly revealed that the healthy wood compositions of the four germplasms were similar, whereas the compositions of the agarwood of the four germplasms were characterized by germplasm-specific features (**Figure 5a**).

As shown in the Venn diagram of the agarwood volatile components in **Figure 5b, 5c**, the agarwood portion of the four germplasms contained a total of 41 identical volatile components, with BMXA containing five unique volatile components, TJA containing eight unique volatile components, and RHA and ASA each containing three unique volatile components. Among the volatile components of the healthy wood layer, there were a total of 18 ordinary components and only 1 to 2 unique volatile components for each group.

**Figure 6:**
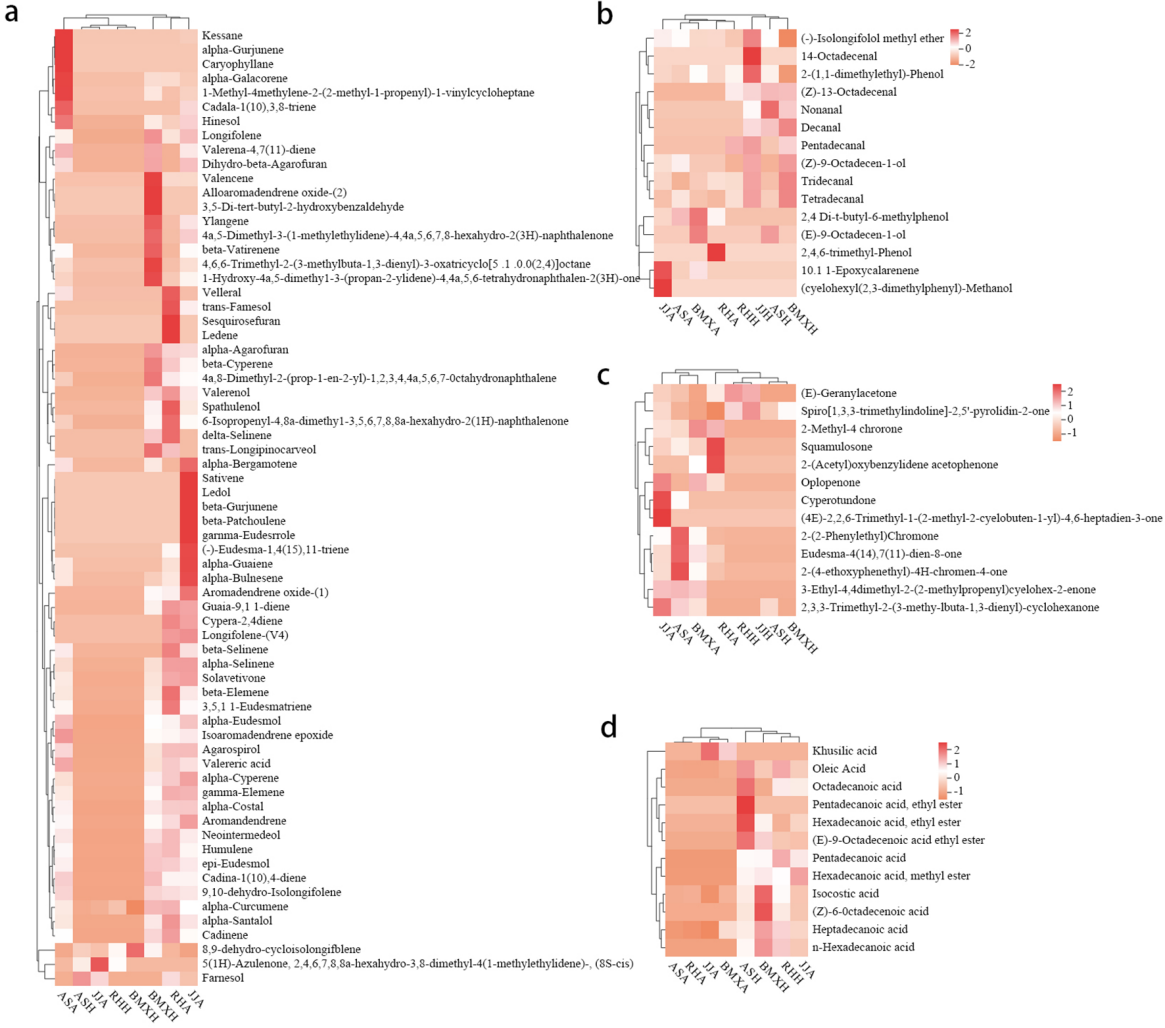
Heatmaps of each component. **(a)** Sesquiterpenes. **(b)** Other aromatic compounds. **(c)** Chromone compounds. **(d)** Fatty acid compounds (n= 3).

**Figure 7:**
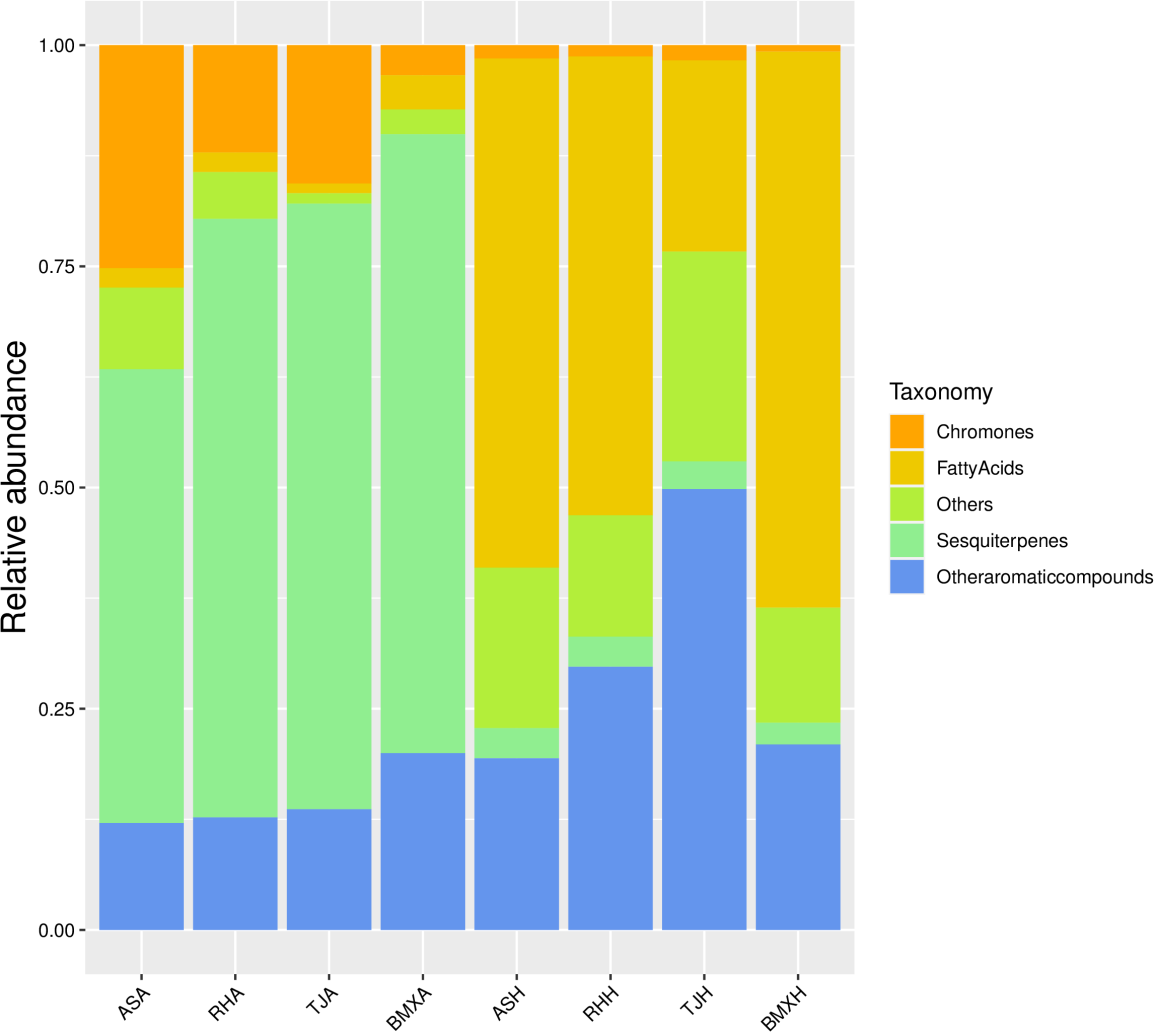
Stacked histograms of the compositional classification of the healthy wood and agarwood layers of the four germplasms of *A. sinensis* (n= 3).

The agarwood layers of each germplasm had many similarities, but there were some differences in the contents of various compounds. According to the analysis of peak area occupancy and a compositional heatmap (**Figure 6**), the ASA germplasm was dominated by 7 sesquiterpene components, such as α-curcumene, kessane, α-calacorene, hinesol, and caryophyllane; BMXA contained mainly valencene, ylangene, β-vatirenene and 15 other sesquiterpenoids; RHA contained 14 main sesquiterpene components, such as velleral, farnesol, and sesquirosefuran; and TJA possessed 16 main sesquiterpene constituents, such as β-gurjunene, β-patchoulene, and γ-eudesmole. After further analysis, we found that the agarwood layer fraction was composed mainly of sesquiterpenoids, small-molecule aromatic substances, chromones and small amounts of fatty acid components. In both the Qinan agarwood and ordinary agarwood, the sesquiterpene components were consistently the most abundant, and the sesquiterpene content in the agarwood layer of the four germplasms reached more than 50%, among which the abundances in TJA, RHA and BMXA were close to 70%, and ASA possessed higher levels of chromones than the other three germplasms. In the agarwood of the three Qinan-type germplasms, the proportion of chromones reached more than 10%, with the highest levels observed in ASA (25%), while the chromone content in the healthy wood of the ordinary-type germplasm was only 3%. Unlike the agarwood layers, the healthy wood layers of the four germplasms had almost no sesquiterpene components. The healthy wood layer was composed mainly of fatty acids and aromatic compounds, of which the fatty acids were the most abundant. The proportion of fatty acids in TJH was approximately 20%. The other three germplasms exhibited a fatty acid proportion of more than 50% in the agarwood layer. Sesquiterpenes and chromones, as the characteristic components of agarwood layer, accounted for only 1%~3% (**Figure 7**). In total, 90 compounds were detected in ordinary agarwood, while 125 compounds were identified in Qinan-type agarwood. Of these, 5 compounds were unique to the agarwood layer of ordinary agarwood, whereas 14 compounds were unique to the agarwood layer of Qinan-type agarwood. The chemical composition of the healthy wood layer was generally similar between the two types. Notably, no unique compounds were detected in the healthy wood layer of ordinary agarwood, whereas the healthy wood layer of Qinan-type agarwood contained 15 unique compounds.

### 3.5 Correlation analysis of endophytic fungi and chemical composition

To analyze the relationships between endophytic fungi and chemical components in agarwood, the top 5 fungal phyla in terms of abundance and the four major classes of compound components in agarwood were selected for correlation analysis. On the basis of the presence of different fungal phyla and chemical components, correlation analysis revealed that fungi in the phylum *Rozellomycota* were correlated with the distribution of aromatic compounds (r=0.720*, p<0.05; **Table 3**). To further determine the correlations between fungal genera and specific compounds, we analyzed the correlations between the 50 most abundant fungal genera in the agarwood layers of different germplasms and the contents of 73 agarwood compounds (**Table 2**). The analysis of sesquiterpenoids revealed a correlation between fungi of the genera *Kurtzmanomyces*, *Fonsecaea*, *Arthopyrenia*, *Trichosporon*, *Fusarium*, *Bipolaris*, and *Apiotrichum* and the formation of sesquiterpenoid compounds in agarwood (r>0.8, p<0.01; **Table 2**). There was a correlation between the abundances of fungi of the genera *Fusarium*, *Hermatomyces*, *Rhinocladiella*, and *Pseudeurotium* and the formation of chromone components in agarwood (r>0.8, p<0.01; **Table 2**). The abundances of *Microidium*, *Cladosporium*, *Cephalotrichum*, *Lysurus* and *Lamelloclavaria* were correlated with the fatty acid content of agarwood. Moreover, the abundances of fungi of the genera Solicoccozyma, Naganishia, and Kurtzmanomyces were correlated with levels of other aromatic compounds in agarwood (r>0.8, p<0.01; **Table 2**). The results of the present analysis may provide a basis for the selection of fungi for use in inducing agarwood formation. Collectively, these results highlight the crucial role of endophytic fungi in influencing the chemical composition of agarwood and lay a solid scientific foundation for developing targeted fungal-based strategies to induce agarwood formation with specific chemical profiles.

**Table 3:** Correlations between volatile compounds and fungal phyla among the four types of agarwood (n= 3).

Fungal phyla Volatile compounds	*Ascomycota*	*Basidiomyota*	*Rozellomycota*	*Chytridiomycota*	*Mortierellomycota*
Sesquiterpenes	-0.535	0.5986	-0.130	0.0361	-0.0003
Fatty acids	0.5267	-0.497	-0.246	0.2070	0.2225
Chromones	-0.580	0.23726	-0.0886	-0.4245	-0.3594
Aromatics	0.3687	-0.2630	0.720*	-0.1059	-0.1013

*Correlation is significant at the 0.05 level (2-tailed).

## 4 Discussion

Endophytes are fungi that colonize living plants and do not cause any immediate, noticeable negative effects (Hyde and Soytong, 2008). Research has revealed that endophytic fungi are widespread and are found in diverse orders, families, and genera of plants. The plant host provides nutrients for endophyte growth and helps the endophytes complete reproduction and transmission; simultaneously, the endophytes help promote host plant growth and development. Research by several scholars has confirmed that the endophytes of certain plants have a growth-promoting effect on host plants (Varga et al., 2020; Shuang et al., 2022; Yuan et al., 2018). Some scholars have also reported that endophytes have antagonistic effects on pathogenic bacteria that cause diseases in host plants and that the presence of such endophytes improves the disease resistance of host plants (Fu et al., 2021). Moreover, some endophytes can improve plant resistance through interactions with host plants, whereas some endophytes can reduce metal toxicity in the interroot zone of plants by distributing metal conjugates to the interroot zone, thus reducing the damage caused by toxic metals to host plants (Domka et al., 2019; Mao et al., 2023). Related studies have confirmed that the presence of endophytes ensures the healthy growth of host plants under nutrient limitation. For example, Tamas Varga et al. reported that endophytic bacteria in two wild poplar varieties contributed to the rerelease of phosphate from poplar trees and promoted the uptake of phosphorus by wild poplar trees (2020). Some scholars have reported that some plant endophytic fungi participate in physiological and biochemical activities within plants by producing secondary metabolites. In conclusion, plant endophytic fungi can form harmonious symbiotic relationships with host plants during long-term evolutionary processes and play crucial roles in plant growth.

In recent years, many studies have confirmed that the reason for the formation of resin is the result of the defense mechanism of ordinary-type *A. sinensis.* Many scholars believe that when the trunk of a tree is subjected to physical and chemical damage or fungal infestation, the tree produces a defensive response, generating signal molecules to induce the production of resin at the injury site to prevent further damage to the tree trunk; this leads to the production of the incense materials used by humans (Sun et al., 2006).

Owing to the low yield of incense produced in natural environments, growers have begun to experiment with artificial methods to induce resin production in healthy white trunks, such as by punching holes or injecting harsh fluids into the trunks. Some of these scholars found that inoculation with different fungi in healthy trunks bearing white rosin can lead to the production of incense with different chemical compositions, which also revealed the correlation between the chemical compositions of incense and fungi. Since then, several researchers have attempted to use different fungi to induce the formation of incense and discovered more species of fungi that can induce incense formation.

In the present study, we analyzed the correlation between the chemical composition of different germplasms of *A. sinensis* and fungi and found that the abundances of some fungi were correlated with the chemical composition of incense, providing more choices for the types of strains that can be used to artificially induce incense formation in the future.

## Data Availability

The original contributions presented in the study are included in the article/**Supplementary Material**. Further inquiries can be directed to the corresponding authors.

## Supplementary material

Supplementary Figure 1 Slices of the four different germplasms of *A. sinensis*: **(a)** ordinary-type germplasm, **(b)** Aoshen germplasm, **(c)** Tangjie germplasm, **(d)** Ruhu germplasm.

Supplementary Table 1 Diversity of fungal germplasms in the eight types of agarwood.
