# Supplementary figures and images for: Combined analysis of the endophytic fungi and volatile oil content of different Aquilaria sinensis germplasms revealed the correlations between endophytic fungal abundances and agarwood production
Source: Front Plant Sci. 2025 May 13;16:1546050. doi: 10.3389/fpls.2025.1546050 (PMC12107704; doi:10.3389/fpls.2025.1546050)

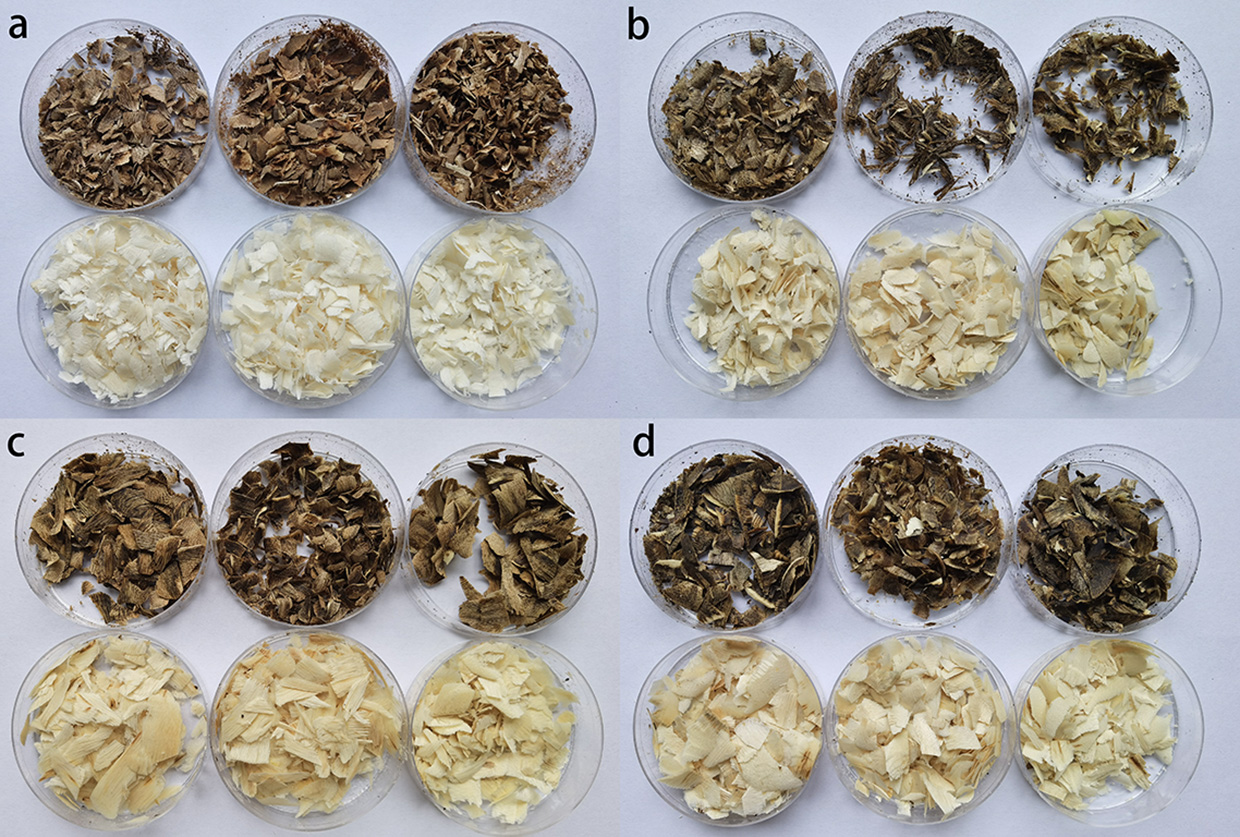

Supplement: Supplementary Figure 1 — Slices of the four different germplasms of A. sinensis: (a) ordinary-type germplasm, (b) Aoshen germplasm, (c) Tangjie germplasm, (d) Ruhu germplasm. [file Image1.jpeg]
