# Supplementary material for: Combined analysis of the endophytic fungi and volatile oil content of different Aquilaria sinensis germplasms revealed the correlations between endophytic fungal abundances and agarwood production
Source: Front Plant Sci. 2025 May 13;16:1546050. doi: 10.3389/fpls.2025.1546050 (PMC12107704; doi:10.3389/fpls.2025.1546050)
Supplement: Supplementary Table 1 — Diversity of fungal germplasms in the eight types of agarwood. [file Table1.doc]

**Table S1.** Diversity of fungal germplasms in the eight types of agarwood.

| Type | Number | Phylum | Class | order | Family | Genus | Germplasm | OTUs |
| --- | --- | --- | --- | --- | --- | --- | --- | --- |
| ASH | 1 | 6 | 26 | 66 | 141 | 227 | 282 | 547 |
|  | 2 | 11 | 34 | 70 | 140 | 220 | 274 | 630 |
|  | 3 | 8 | 30 | 64 | 125 | 188 | 227 | 453 |
| ASA | 1 | 10 | 37 | 79 | 147 | 227 | 281 | 607 |
|  | 2 | 9 | 32 | 72 | 134 | 203 | 247 | 416 |
|  | 3 | 5 | 27 | 63 | 130 | 199 | 240 | 484 |
| TJH | 1 | 9 | 28 | 60 | 114 | 178 | 215 | 480 |
|  | 2 | 11 | 32 | 79 | 168 | 244 | 307 | 765 |
|  | 3 | 7 | 31 | 78 | 152 | 232 | 286 | 592 |
| TJA | 1 | 5 | 17 | 35 | 61 | 76 | 93 | 178 |
|  | 2 | 8 | 25 | 53 | 86 | 108 | 124 | 293 |
|  | 3 | 6 | 22 | 48 | 74 | 87 | 106 | 214 |
| RHH | 1 | 9 | 28 | 60 | 114 | 151 | 179 | 372 |
|  | 2 | 11 | 38 | 86 | 169 | 255 | 322 | 645 |
|  | 3 | 10 | 30 | 81 | 161 | 251 | 311 | 622 |
| RHA | 1 | 10 | 32 | 81 | 162 | 241 | 304 | 638 |
|  | 2 | 8 | 25 | 57 | 104 | 163 | 201 | 402 |
|  | 3 | 6 | 20 | 47 | 85 | 121 | 144 | 306 |
| BMXH | 1 | 8 | 26 | 60 | 118 | 176 | 206 | 470 |
|  | 2 | 11 | 32 | 72 | 141 | 225 | 277 | 593 |
|  | 3 | 10 | 32 | 71 | 149 | 226 | 271 | 634 |
| BMXA | 1 | 10 | 25 | 56 | 115 | 181 | 222 | 556 |
|  | 2 | 9 | 28 | 65 | 119 | 177 | 211 | 431 |
|  | 3 | 11 | 33 | 80 | 158 | 238 | 284 | 588 |
